# Constitutive Activation of the Nlrc4 Inflammasome Prevents Hepatic Fibrosis and Promotes Hepatic Regeneration after Partial Hepatectomy

**DOI:** 10.1155/2015/909827

**Published:** 2015-11-09

**Authors:** David A. DeSantis, Chih-Wei Ko, Lan Wang, Peter Lee, Colleen M. Croniger

**Affiliations:** Department of Nutrition, Case Western Reserve University School of Medicine, Cleveland, OH 44106, USA

## Abstract

TThe molecular mechanisms responsible for the development of hepatic fibrosis are not fully understood. The Nlrc4 inflammasome detects cytosolic presence of bacterial components, activating inflammatory cytokines to facilitate clearance of pathogens and infected cells. We hypothesized that low-grade constitutive activation of the Nlrc4 inflammasome may lead to induced hepatocyte proliferation and prevent the development of hepatic fibrosis. The gene of Nlrc4 contains two single nucleotide polymorphisms (SNPs), one located within the Nlrc4 promoter and one contained within exon 5. These SNPs regulate Nlrc4 gene transcription and activation as measured through gene reporter assays and IL-1*β* secretion. The 17C-6 mice have increased IL-1*β* in plasma after chronic carbon tetrachloride (CCl_4_) administration compared to B6 mice. After two-thirds partial hepatectomy (2/3PH) 17C-6 mice have earlier restoration of liver mass with greater cyclin D1 protein and BrdU incorporation compared to B6 mice at several time points. These data reveal mild constitutive activation of the Nlrc4 inflammasome as the results of two SNPs, which leads to the stimulation of hepatocyte proliferation. The increased liver regeneration induces rapid liver mass recovery after hepatectomy and may prevent the development of hepatotoxin-induced liver fibrosis.

## 1. Introduction

Nonalcoholic fatty liver disease (NAFLD) has become a significant cause of chronic liver disease with staggering occurrence in the USA and worldwide [1]. NAFLD disease prevalence studies estimate anywhere between 2.8% and 38.5% of the US general population has NAFLD [2–6]. Epidemiological studies indicate NAFLD is pathophysiologically linked to metabolic syndrome as it is associated with obesity, hypertension, dyslipidemia, and insulin resistance [7, 8]. Current approximations have greater than 75% of obese individuals afflicted with NAFLD [9].

NAFLD is a term used to label a spectrum of liver diseases ranging from early stage fatty liver (steatosis) to advanced cirrhosis of the liver and hepatocellular carcinoma [10]. This disease is aptly named as it occurs in individuals who consume little to no alcohol and the NAFLD pathology closely resembles that of a diseased liver attributable to alcohol abuse [11]. NAFLD is believed to originate with the accumulation of fatty acids within the liver as a result of insulin resistance [12]. Consequently increased membrane lipid peroxidation and oxidative stress within the cells of the liver result in inflammation and increased deposition of extracellular matrix (ECM) proteins, that is, fibrosis [13]. Excessive fibrosis leads to atypical hepatic arrangement and subsequent scarring. As tissue scarring develops there is decreased hepatic blood flow which initiates hepatocellular dysfunction [14]. Genetic factors contribute to an individual's predisposition to the development and progression of NAFLD [15].

The development of hepatic fibrosis is regulated primarily by hepatic stellate cells (HSCs), which synthesize ECM proteins. In a quiescent state, HSCs store vitamin A as retinol esters and make up roughly one-third of nonparenchymal cells of the liver. When HSCs are activated they become fibrogenic. HSCs are activated by injured hepatocytes and stimulated resident macrophages known as Kupffer cells (KC) [16, 17]. KC play a significant role in immune surveillance and production of cytokines such as tumor necrosis factor *α* (TNF-*α*), interleukin-1*β* (IL-1*β*), and interleukin-6 (IL-6) [18]. Interestingly KC have been shown to be essential for liver regeneration after partial hepatectomy of the liver [19] via cytokine production [20, 21]. KC and their immune response to various toxic insults play a role in disease progression and hepatic homeostasis [22].

The inflammasome is a multimeric protein complex that is assembled and activated upon the detection of cellular infection or cell stress [23]. Damage-associated molecular patterns (DAMP) and pathogen-associated molecular patterns (PAMP) bind to pattern-recognition receptors inducing an intracellular signaling cascade. These events lead to oligomerization of inflammasome components, activation of intracellular cysteine protease caspase-1, and the subsequent cleavage and maturation of proinflammatory cytokines IL-1*β* and IL-18 [24–27].

The Nlrc4 inflammasome detects the cytosolic presence of bacterial flagellin during infection using NLR family, apoptosis inhibitory protein 5 (Naip5), and NLR family, CARD domain containing 4 (Nlrc4) to form a heterooligomeric inflammasome structure [28, 29]. Several activated Nlrc4s then form an inflammasome complex by which Nlrc4 uses an N-terminal caspase activating and recruitment domain (CARD) to interact with the CARD of pro-caspase-1, leading to cleavage and activation of caspase-1. The Nlrc4 inflammasome may associate with caspase-1 independent of adaptor protein apoptosis-associated speck-like protein containing a caspase recruitment domain (ASC) which itself contains a CARD [30].

Identification of genetic factors contributing to the pathogenesis of NAFLD facilitates the potential to target susceptible individuals for interventional strategies to ameliorate NAFLD progression. Genome-wide association studies (GWAS) are an unbiased tool for the identification of gene variants associated with genetic traits. Unfortunately, GWAS examining phenotypes relevant to NAFLD are lacking. A 2010 GWAS by Chalasani et al. linked genes involved in lipid metabolism and collagen deposition with NAFLD characterized by histology [31]. A 2015 GWAS found the Nlrc4 inflammasome was involved in IL-18 production in patients with acute coronary syndromes [32]. Association studies such as these should be used in conjunction with hypothesis-driven investigative studies to identify key genetic regulators of NAFLD. We have previously shown allelic differences of the Nlrc4 gene between inbred genetic mouse strains C57BL/6J (B6) and A/J modulate the development and/or resolution of hepatic fibrosis, a critical stage of NAFLD development [33]. Identifying the molecular mechanisms by which Nlrc4 modulates liver fibrosis is essential to fully understand the complex dynamics of liver disease origin and development. Using models of hepatotoxin-induced liver injury and liver regeneration after partial hepatectomy, we established the role of Nlrc4 in governing hepatic fibrosis development.

## 2. Materials and Methods

### 2.1. Animal Husbandry

All procedures involving animals were approved by the Case Western Reserve University Institutional Animal Care and Use Committee. B6 and A/J mice were obtained from Jackson Laboratories and maintained at Case Western Reserve University for over 10 generations [34]. Congenic strain 17C-6 was derived from CSS-17 as previously described [35]. Mice were housed in a microisolator environment on a 12 hr : 12 hr light/dark cycle. All mice were weaned at 3-4 weeks of age and maintained on LabDiet #5010 autoclavable rodent chow (LabDiet, Richmond, IN) with food and water provided* ad libitum*.

### 2.2. Carbon Tetrachloride (CCl_4_) Administration and Tissue Collection

For both acute and chronic CCl_4_ studies, CCl_4_ (Sigma-Aldrich, St. Louis, MO) was performed as previously described [36]. Mice were sacrificed at 24 hours and 48 hours after injection. All control mice receiving olive oil were sacrificed 48 hours after injection. For chronic CCl_4_ administration, mice were given two injections weekly (Tuesday/Friday) for 5 weeks. Mice were gradually increased in dose of CCl_4_ over the first three injections (first, 0.25 *μ*L/g body weight; second, 0.5 *μ*L/g body weight; third and subsequent doses, 1 *μ*L/g body weight). Both CCl_4_ and olive oil treated mice were sacrificed 72 hours after the final injection. Mice were allowed access to food and water* ad libitum* for the duration of the study. Blood plasma and liver tissue were collected and frozen until further processing.

### 2.3. DNA Constructs, Cell Transfections, and Reporter Assays

DNA was isolated from B6 and A/J mice. Using custom designed primers that span the 1000 bp region preceding the start site of transcription for Nlrc4, we isolated the Nlrc4 promoter by polymerase chain reaction (PCR). Each 1 kb promoter was cloned into a pSC-A-amp/kan cloning vector using StrataClone PCR cloning kit (Agilent Technologies, Santa Clara, CA) and then subcloned into the luciferase plasmid vector pGL4.10-luc2 using QuickLink DNA Ligation Kit (Sigma-Aldrich, St. Louis, MO) and sequenced by the Case Western Reserve University Genomics Core. The expression constructs were cotransfected with either an overexpression vector for Cdx-1 driven by cytomegalovirus (CMV) promoter or a control vector containing only the CMV promoter (Open Biosystems Products, Huntsville, AL) into murine macrophage cell line RAW 264.7 using FuGENE HD Transfection Reagent (Promega, Madison, WI). Cells were cultured using ATCC-formulated Dulbecco's Modified Eagle's Medium (Life Technologies, Grand Island, NY) supplemented with 10% fetal bovine serum (Life Technologies, Grand Island, NY). Dual-Glo Luciferase Assay System (Promega, Madison, WI) activating firefly (*Photinus pyralis*) and Renilla (*Renilla reniformis*) luciferases was used to control for transfection efficiency. Luminescent signal was measured by plate-reading illuminometer (Molecular Devices, Sunnyvale, CA) and activity was calculated according to the manufacturer's protocol.

### 2.4. Chromatin Immunoprecipitation

ChIP was conducted on a cultured murine macrophage cell line RAW 264.7 as previously described [37]. The cells were cross-linked with 11% buffered formaldehyde solution and sonicated into DNA fragments. DNA fragments were selected using a Cdx-1 antibody (Abcam, Cambridge, MA) conjugated with magnetic beads and purified. The fragments were analyzed by PCR (Life Technologies, Grand Island, NY). Custom designed primers (IDT, Coralville, IA) were used to detect potential transcription factor Cdx-1 binding sites (Supplemental Table 1 in Supplementary Material available online at http://dx.doi.org/10.1155/2015/909827). Anti-GFP antibody (Abcam, Cambridge, MA) served as an antibody negative control. Primers for a negative control locus and a nontemplate control were used during RT-PCR. The amplified products were visualized on 2% agarose gels.

### 2.5. Bone Marrow-Derived Macrophages (BMDM)

12–16-week-old male B6 and congenic 17C-6 mice were anesthetized and their hind legs harvested from the pelvis keeping the femur bone intact. The surrounding skin and muscle tissues were removed revealing femur, tibia, and fibula bones. In an aseptic environment the femur and tibia bones were cut at each end and the lumen was flushed with culture media. The marrow cells obtained were strained, purified, and plated. Cells were cultured in macrophage differentiation medium containing 10 ng/mL macrophage colony-stimulating factor (M-CSF) at 37°C in an air atmosphere with 5% CO_2_ for 7–10 days to differentiate macrophage progenitor cells into mature macrophages as described [38, 39].

Once BMDM were fully differentiated they were exposed to bacterial LPS (100 ng/mL) for 2, 4, and 8 hours at 37°C in an air atmosphere with 5% CO_2_. Culture media were collected and cells were then washed in 1x PBS and collected. Subcellular fractionation of BMDM cells was prepared as previously described and either RNA or protein was isolated [40]. To test the role of caspase-1, we used 250 *μ*M selective irreversible caspase-1 inhibitor, acetyl-Tyr-Val-Ala-Asp-chloromethylketone (Ac-YVAD-cmk) (Sigma-Aldrich, St. Louis, MO). The BMDM were isolated and incubated with the caspase-1 inhibitor YVAD (50 *μ*M) for 0.5 hours. The BMDM were then stimulated with LPS (200 ng/mL) for 4 hours while still exposed to the inhibitor and then treated with 1 mM ATP for an additional 0.5 hours. After inhibitor incubation, LPS priming, and ATP incubation the cell-free media sample was collected and cells were washed twice with cold 1x PBS and then processed for analysis of protein and gene expression.

### 2.6. Stimulation of Macrophages with Flagellin

Lipofectin Reagent was purchased from Thermo Fisher (Grand Island, NY) and used according to the manufacturer's instructions. Briefly, Lipofectin Reagent was prepared and incubated with bovine serum albumin as a control (500 ng/mL) for 24 hours or flagellin (500 ng/mL) for 24 hours. Macrophages were then stimulated with ATP (5 mM) for 0.5 hours. At the conclusion of the experiment cell media were collected and cells were lysed and the cell supernatant was collected. IL-1*β* was measured by ELISA (BioLegend, San Diego, CA) according to manufacturer's instructions.

### 2.7. 2/3 Partial Hepatectomy

12–16-week-old male B6 and congenic 17C-6 mice underwent two-thirds partial hepatectomy surgery performed by Case Western Reserve University Mouse Metabolic Phenotyping Center (U24-DK76174) as previously described [41]. The weight of resected liver for each mouse was recorded at the time of surgery. We found that resection of the median and left lateral lobes resulted in an average removal of ≈55% of total liver tissue. The animals recovered for 2-, 4-, 8-, 12-, 36-, and 168-hour (7 days) time periods and then were euthanized. Blood plasma was taken and the remaining liver tissue was removed, weighed, and frozen. The animal survival rate was >95% at all time points with no statistical difference between genotypes. Percent liver regeneration was calculated by dividing the weight of the liver at the time of sacrifice by the initial liver weight of animal and multiplying by 100. The initial liver weight was obtained by assuming the resected liver weight was 55% of original liver mass [42, 43]:

 % liver regeneration = (*A*/*B*) × 100,
 where *A* is liver weight at sacrifice,
 
*B* is estimated liver weight before PH,
 
*B* = (resected liver during PH)/0.55.

### 2.8. RNA Isolation, cDNA Synthesis, and Real-Time Quantitative PCR (qPCR)

Total RNA from 30 mg of liver tissue was isolated from B6 and 17C-6 mice after 2/3 partial hepatectomy using NucleoSpin RNA Kit (Macherey-Nagel, Bethlehem, PA). cDNA was synthesized from 500 ng total RNA using random hexamer primers and MMTV reverse transcriptase (Applied Biosystems, Foster City, CA). Real-Time qPCR analysis was performed using Bullseye EvaGreen SYBR qPCR reagent (MidSci, St. Louis, MO) on a Chromo4 Cycler (MJ Research/Bio-Rad, Hercules, CA). Primer sequences were custom designed using Primer3Plus website version 2.3.6 (http://www.bioinformatics.nl/primer3plus/) (Supplemental Table 2). Endogenous housekeeping control 18S rRNA was used to account for load variation. Data was normalized by comparative Ct method. A ΔΔCt value was obtained by subtracting control ΔCt values from experimental ΔCt values. The ΔΔCt value is converted to fold difference compared to endogenous housekeeping control by raising two to the −ΔΔCt [35, 44].

### 2.9. Plasma Alanine Aminotransferase

The levels of ALT in plasma of B6 and 17C-6 mice after 2/3 partial hepatectomy were measured using a commercially available enzymatic assay kit (Sekisui Diagnostics, Lexington, MA) as per manufacturer's directions.

### 2.10. Protein Isolation and Western Blotting

Whole cell protein isolation from B6 and 17C-6 mice after 2/3 partial hepatectomy was performed as previously described [45]. Polyvinyl difluoride membranes were incubated with an antibody for cyclin D1 (1 : 1,000; Santa Cruz Biotechnology, Dallas, TX), for STAT3 and phosphorylated STAT3 (Tyr^705^) (1 : 1,000; Santa Cruz Biotechnology, Dallas, TX). Immunoreactive proteins were measured by scanning densitometry (UN-SCAN-IT software, Orem, UT). Membranes were stripped using ReView Buffer Solution (Amresco, Solon, OH). Western blots were normalized for loading differences using heat shock cognate protein 70 (HSC-70) (1 : 16,000; Santa Cruz Biotechnology, Dallas, TX) as previously described [45].

### 2.11. IL-1*β*, IL-18, and IL-6 ELISA

Blood plasma isolated from B6 and 17C-6 mice after 2/3 partial hepatectomy was used to measure IL-1*β* (BioLegend, San Diego, CA), IL-18 (eBioscience, San Diego, CA), and IL-6 (eBioscience, San Diego, CA) by ELISA according to manufacturer's directions. The IL-1*β* (BioLegend, San Diego, CA) ELISA was also used for BMDM cell lysis supernatant discussed above.

### 2.12. BrdU Immunohistochemical Analysis and Microscopy

B6 and 17C-6 mice that had undergone 2/3 partial hepatectomy were intraperitoneally injected (IP) with bromodeoxyuridine (BrdU) labeling reagent (Life Technologies, Grand Island, NY) 2 hours before sacrifice. Freshly dissected liver was fixed in 10% neutral buffered formalin solution containing 4% formaldehyde for 48 hours and then embedded in paraffin and sectioned to 5 *μ*m and slide mounted. Sections were deparaffinized, rehydrated using a series of graded alcohol solutions to 80%, and then trypsin digested. BrdU staining procedure was done using BrdU staining kit (Life Technologies, Grand Island, NY) according to kit manufacturer's recommended instructions. Slides were counterstained using hematoxylin. Images were obtained using a Leica DM6000 upright microscope (Case Western Reserve University Imaging Core Facility, Department of Genetics and Genome Sciences, Case Western Reserve University) and Velocity Acquisition Software (PerkinElmer, Waltham, MA). Four high-powered zones representative of slides were used to calculate positive BrdU incorporation.

### 2.13. Statistical Analysis

The statistical differences reported are means ± standard error of the mean (SEM). Statistics were calculated by unpaired Student's *t*-test or by two-way ANOVA with Bonferroni correction for multiple testing using GraphPad Prism 5.0 software (GraphPad, San Diego, CA).

## 3. Results

### 3.1. Cdx-1 Binds to the Mouse Nlrc4 Promoter

**Figure 1:**
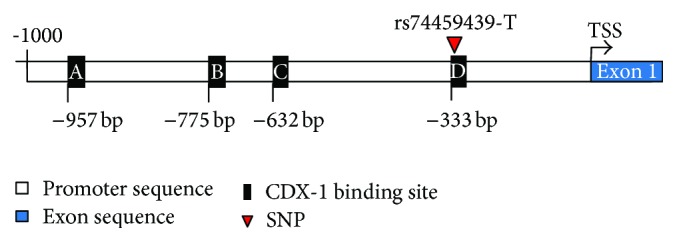
Nlrc4 promoter and Cdx-1 binding sites. Graphical depiction of 1000 bp promoter region preceding the transcriptional start site (TSS) of gene Nlrc4. Potential transcription factor Cdx-1 interaction sites (as detected by TFSEARCH application) are labeled in black boxes designated A (−957 bp), B (−775 bp), C (−632 bp), and D (−333 bp). Genetic single nucleotide polymorphism (SNP) rs74459439-T is located −331 bp upstream of the TSS labeled as red triangle. Exon 1 of the Nlrc4 gene is labeled in blue.

Gene sequencing analysis of the B6 and A/J Nlrc4 promoter revealed a SNP 331 bp upstream of the transcriptional start site (Figure 1). The A/J promoter had a single thymine deletion at this site (rs74459439-T). Transcription factor binding site analysis software (TFSearch Internet-Based Tool) associated the location of this polymorphism as having a high likelihood for transcription factor Cdx-1 binding (TFSEARCH score of 92.1).

### 3.2. Cdx-1 Governs Nlrc4 Gene Expression in Mouse Macrophages

**Figure 2:**
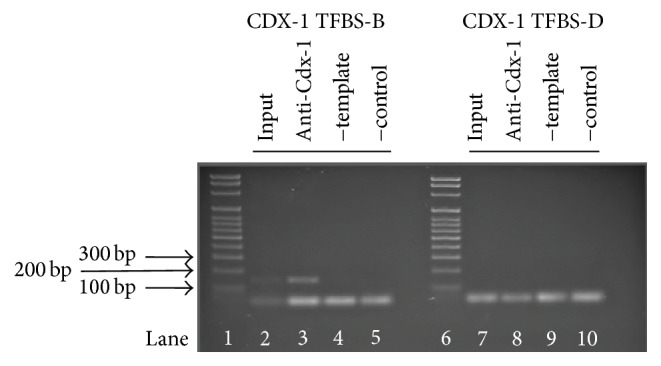
Cdx-1 binds to the mouse Nlrc4 promoter. ChIP technique verifies that Cdx-1 interacts with Nlrc4 promoter, but not with site TFBS-D containing SNP rs74459439-T. ChIP analysis of DNA in cross-linked chromatin from RAW 264.7 cells. DNA fragments were precipitated using an anti-Cdx-1 antibody. Lanes 2–5: primer sequence targeting TFBS-B using RT-PCR. Lanes 7–10: primer sequence targeting TFBS-D using RT-PCR. Lane 1: DNA Ladder. Lane 2: 1% starting chromatin input. Lane 3: DNA precipitated with anti-Cdx-1 antibody. Lane 4: nontemplate control. Lane 5: negative control using anti-GFP antibody. Lane 6: DNA Ladder. Lane 7: 1% starting chromatin input. Lane 8: DNA precipitated with anti-Cdx-1 antibody. Lane 9: nontemplate control. Lane 10: negative control using anti-GFP antibody. RT-PCR products were visualized using a 2% agarose gel.

**Figure 3:**
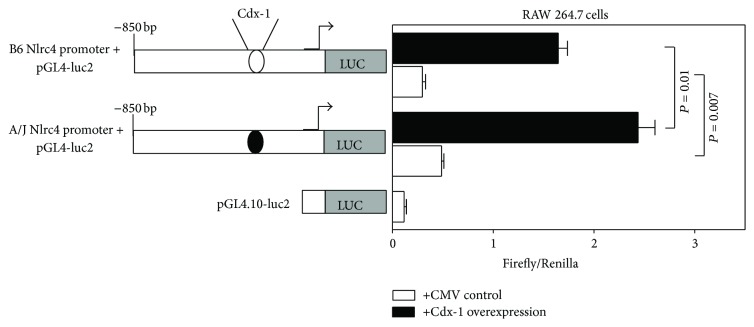
Cdx-1 governs Nlrc4 gene expression in murine macrophage cell line RAW 264.7. DNA spanning 850 bp upstream of the transcriptional start site from gene Nlrc4 was amplified and isolated from B6 and A/J mice. These fragments were separately cloned into a pSC-A-amp/kan cloning vector and then subcloned into luciferase plasmid vector pGL4.10-luc2. Constructs were cotransfected with either an overexpression vector for Cdx-1 driven by a CMV promoter or the CMV promoter for a control. Luminescence of gene reporter firefly (*Photinus pyralis*) and transfection efficiency reporter Renilla (*Renilla reniformis*) was quantified. White bars represent relative firefly luminescence of Nlrc4 promoter cotransfected with CMV promoter only. Black bars represent relative firefly luminescence of Nlrc4 promoter vector cotransfected with Cdx-1 overexpression vector driven by CMV promoter. B6 Nlrc4 promoter indicates an 850 bp sequence containing the B6 version of SNP rs74459439-T at location −331 bp. A/J Nlrc4 promoter indicates an 850 bp sequence containing the A/J version of SNP rs74459439-T at location −331 bp. Values represent the mean ± SEM. Statistics were calculated by unpaired Student's *t*-test, *n* = 4/group.

To definitively establish Cdx-1 binding to the Nlrc4 promoter, Chromatin Immunoprecipitation (ChIP) analysis was performed on RAW 264.7 cells (B6 allele of the Nlrc4 promoter) using primer sets spanning the SNP, rs74459439-T. Chromatin Immunoprecipitation (ChIP) analysis revealed transcription factor Cdx-1 binds to the mouse Nlrc4 promoter at 3 potential Cdx-1 binding sites but not at the region of the SNP (rs74459439-T) (Figure 2, Supplemental Figure 1). To investigate whether this SNP results in Nlrc4 expression differences we transfected pGL4.10 [luc2] luciferase vectors with the 1000-nucleotide promoter sequence from either the B6 or A/J allele, encompassing this SNP. These constructs were independently cotransfected with an overexpression vector for transcription factor Cdx-1 driven by CMV promoter or the CMV plasmid alone for control into murine RAW 264.7 cells (Figure 3). Both the B6 and A/J Nlrc4 promoter constructs had basal expression of luciferase activity and the luciferase activity was increased when Cdx-1 was overexpressed. More importantly, the A/J allele shows a statistically higher luciferase activity than B6 with Cdx-1 overexpression (Figure 3). These results indicate not only that Cdx-1 is playing a role in Nlrc4 gene expression with murine macrophages, but also that there is a difference in its gene regulatory capacity contingent on the SNP (rs74459439-T) version it contains.

### 3.3. 17C-6 Macrophages Display Increased Inflammasome Activity

Previously, deep sequencing analysis of the B6 and A/J mouse 17th chromosome revealed a SNP (rs29502769) within exon 5 of Nlrc4 resulting in the missense mutation I756V [32]. We identified a new SNP (rs74459439-T) in the promoter of Nlrc4 (Figure 1).

**Figure 4:**
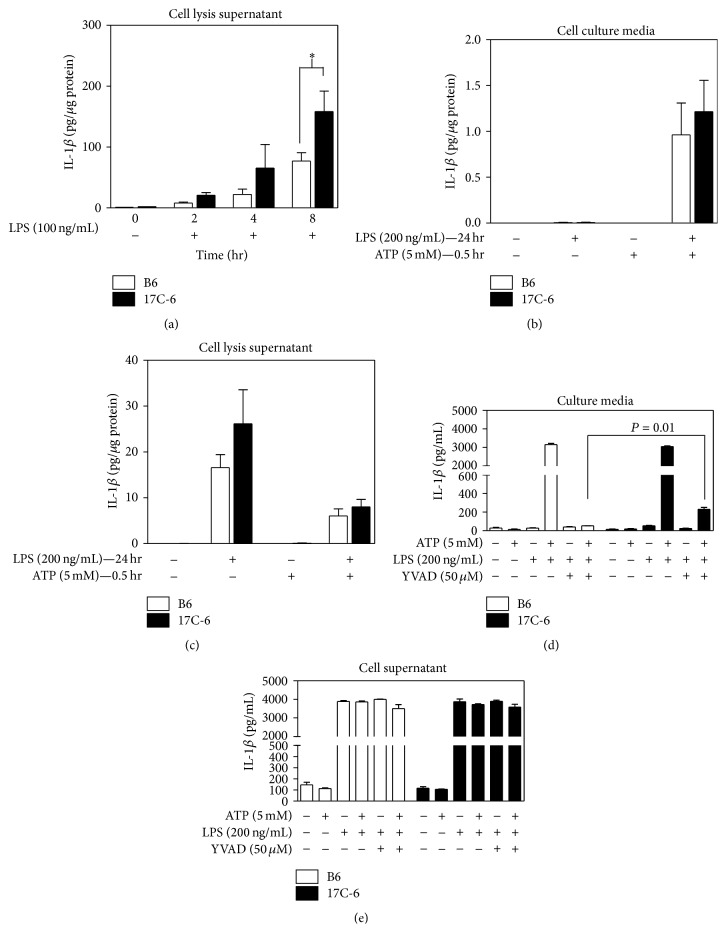
Bone marrow-derived macrophages from 17C-6 congenic mouse produce more IL-1*β* over time. Macrophages were isolated from the bone marrow of 12–16-week-old male B6 and 17C-6 mice. Isolated macrophages were purified and differentiated in culture to mature macrophages using macrophage differentiation media containing M-CSF (10 ng/mL) for 7–10 days. (a) Mature macrophages were exposed to LPS (100 ng/mL) for 2-, 4-, or 8-hour periods. Unexposed cells served as control. Cells were then lysed and the cell lysis supernatant was obtained and IL-1*β* concentration was quantified by ELISA. (b) Mature macrophages were exposed to LPS (200 ng/mL) for 24 hours and then stimulated with 5 mM ATP for 0.5 hours. Cell culture media were collected and (c) cells were lysed to obtain the cell lysis supernatant and IL-1*β* concentrations were quantified by ELISA. (d) The BMDM were isolated and treated with the caspase-1 inhibitor YVAD (50 *μ*M) for 0.5 hours. They were then stimulated with LPS (200 ng/mL) for 4 hours followed by 5 mM ATP for an additional 0.5 hours. After inhibitor incubation, LPS priming, and ATP incubation the cell-free media sample was collected and (e) cells were lysed and the cell lysis supernatant was isolated. IL-1*β* concentrations were quantified by ELISA. Values represent the mean ± SEM. ^*∗*^ *P* < 0.05. Statistics were calculated by two-way ANOVA and Bonferroni correction for multiple testing, *n* = 3–6/group.

To determine the effect these SNPs have on Nlrc4 inflammasome activity, we measured the end product of the Nlrc4 inflammasome, IL-1*β*. BMDM were isolated, differentiated, and cultured. The BMDM were stimulated with bacterial lipopolysaccharide (LPS) (100 ng/mL) for 2, 4, and 8 hours. After incubation, media and cells were isolated. The 17C-6 BMDM containing the A/J allele of Nlrc4 have increased cellular IL-1*β* compared to B6. After 8 hours the difference was statistically significant (Figure 4(a)). When 17C-6 BMDM are exposed to LPS (200 ng/mL) for 24 hours they secrete equal amounts of IL-1*β* into the media as B6 BMDM (Figure 4(b)). Looking inside the BMDM cell using the cell lysis supernatant after LPS for 24 hours, the B6 and 17C-6 BMDM have equivalent concentrations (Figure 4(c)). When stimulated with ATP, IL-1*β* secretion was comparable between B6 and 17C-6 demonstrating there are likely no differences in IL-1*β* secretion biomechanics.

It is possible that the Nlrc4 inflammasome plays a role in inflammatory activity independent of normal inflammasome activation and subsequent caspase-1 cleavage/activation [46, 47]. In fact, the data in Figures 4(d) and 4(e) indicate that Nlrc4 may have a caspase-1 independent maturation and secretion of IL-1*β*. Using a selective, irreversible inhibitor of caspase-1, Ac-YVAD-cmk, we observed a measurable increase in IL-1*β* secretion into the cell media in BMDM derived from 17C-6 mice when compared to B6 mice. When caspase-1 was pretreated with its inhibitor, any subsequent IL-1*β* maturation and secretion would be derived in a non-caspase-1 mediated fashion. This implicates the Nlrc4 inflammasome in a mechanism of IL-1*β* processing independent of caspase-1.

### 3.4. Chronic CCl_4_ Treatment: 17C-6 Mice Secrete Elevated IL-1*β* into the Plasma

**Figure 5:**
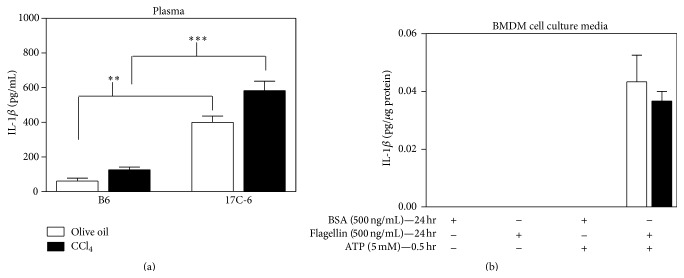
17C-6 congenic mice have increased plasma IL-1*β* after chronic CCl_4_ that appears independent of Nlrc4 stimuli bacterial flagellin. (a) B6 and 17C-6 mice were given an intraperitoneal injection of hepatotoxin CCl_4_ or olive oil vehicle for 5 weeks. Mice were sacrificed 72 hours after their final injection and their blood plasma was collected. Plasma IL-1*β* was measured by ELISA. (b) Lipofectin Reagent was prepared and incubated with bovine serum albumin (BSA) (500 ng/mL) as a control or flagellin (500 ng/mL) for 24 hours; then the macrophages were stimulated with ATP (5 mM) for 0.5 hours. At the conclusion of the experiment cell media were collected and IL-1*β* was measured by ELISA. Values represent the mean ± SEM. ^*∗∗*^ *P* < 0.01. ^*∗∗∗*^ *P* < 0.001. Statistics were calculated by unpaired Student's *t*-test, *n* = 3-4/group.

Mice with the A/J allele of Nlrc4 are resistant to hepatotoxin CCl_4_-induced liver fibrosis [33], therefore, we hypothesize that Nlrc4 may modulate the development of fibrosis. To determine if the function of Nlrc4 impacts this process, we measured IL-1*β* in B6 and 17C-6 mice after chronic exposure (5 weeks) to CCl_4_. We found that B6 mice have roughly a 3.3-fold increase in plasma IL-1*β* after chronic CCl_4_ when compared to B6 olive oil injected control. Congenic strain 17C-6, which has the A/J allele for Nlrc4, had 1.3-fold increase over 17C-6 olive oil control. In addition, congenic 17C-6 mice had increased IL-1*β* compared to B6 mice both for olive oil control (12.9-fold) and after chronic CCl_4_ exposure (5.2-fold) (Figure 5(a)). Thus, 17C-6 mice have constitutive activation of the Nlrc4 inflammasome resulting in an increase of IL-1*β* maturation and secretion into the plasma. To determine if the constitutive release of IL-1*β* requires the stimulation with NLRC4 ligand, flagellin, we analyzed differentiated BMDM treated with bovine serum albumin (BSA) as a control (500 ng/mL) or flagellin (500 ng/mL) for 24 hours; then the macrophages were stimulated with ATP (5 mM) for 0.5 hours (Figure 5(b)). At the conclusion of the experiment cell culture media were collected and IL-1*β* was measured by ELISA. There were no differences in the secretion of IL-1*β* with flagellin suggesting that a noncanonical pathway is contributing to the increase of IL-1*β*.

### 3.5. Partial Hepatectomy Experiments: 17C-6 Mice Show Increased Regenerative Capacity after 2/3 Partial Hepatectomy

**Figure 6:**
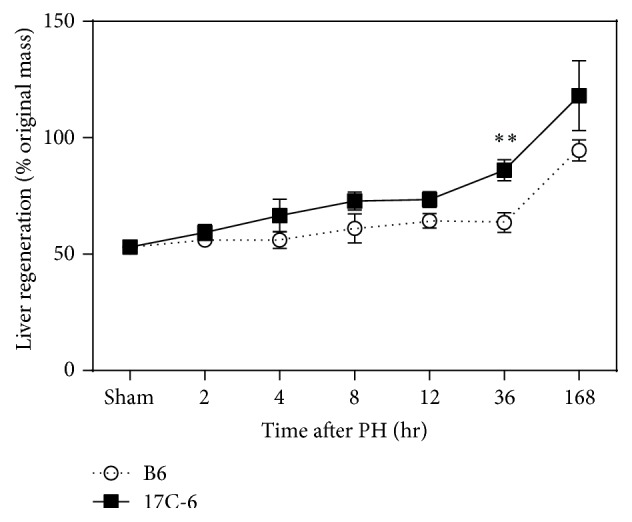
17C-6 mice have increased regenerative capacity after 2/3 partial hepatectomy. Two-thirds PH surgeries were performed on 17C-6 congenic and B6 mice. Percent liver regeneration was calculated by dividing the liver weight at sacrifice by the initial liver weight and multiplying by 100. Initial liver weight was determined assuming the resected liver weight was ~55% of original liver mass. Data is statistical for the source of variation across genotype (*P* < 0.0001) and time points (*P* < 0.0001). Values represent the mean ± SEM. ^*∗∗*^ *P* < 0.01. Statistics were calculated by two-way ANOVA and Bonferroni correction for multiple testing, *n* = 4-5/group.

To fully test the livers ability to proliferate after injury a 2/3PH was performed on B6 and 17C-6 mice. Time points chosen after 2/3PH were 2, 4, 8, 12, 36, and 168 hours after surgery. B6 and 17C-6 mice euthanized immediately after sham surgery showed 55% of original liver mass was remaining; thus this is the starting point of percent original mass (Figure 6). By 2 hours 17C-6 had recovered to 59% of original liver mass, while B6 recovered to 56%. The divergence between the two groups becomes significant at 36 hours after surgery with 17C-6 animals averaging 87% original liver mass, while B6 animals average 57% of original liver mass (Figure 6). Both genotypes fully restored their initial liver mass by 7 days (168 hours) after 2/3PH surgery.

**Figure 7:**
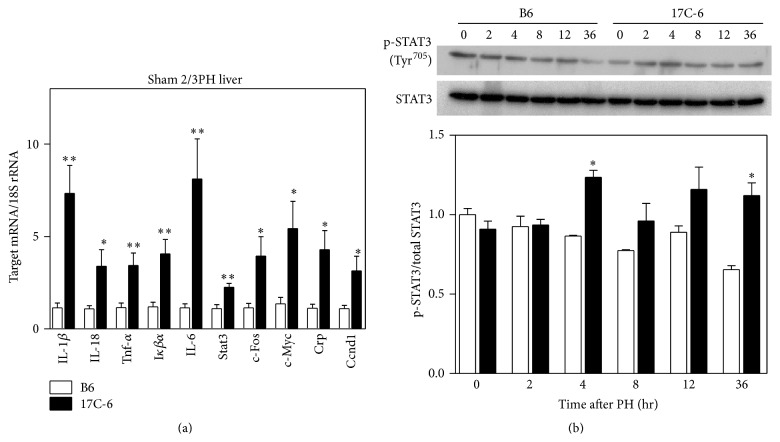
17C-6 mice have increased mRNA of inflammation and cellular proliferation genes after sham 2/3 partial hepatectomy. (a) Sham 2/3PH surgeries were performed on 17C-6 congenic and B6 mice. Total RNA was isolated from liver tissue. Target gene mRNA was quantified by qPCR analysis and normalized with 18S rRNA. (b) 2/3PH surgeries were performed on 17C-6 congenic and B6 mice. Protein was isolated and analyzed by Western blot analysis for expression of STAT-3 and phosphorylated STAT-3. Densitometric quantification of Western blot analysis is graphed with a representative Western blot image. Values represent the mean ± SEM. ^*∗*^ *P* < 0.05. ^*∗∗*^ *P* < 0.01. Statistics were calculated by unpaired Student's *t*-test, *n* = 4–6/group.

It has been well established that TNF-*α*, IL-1*β*, and IL-6 activate transcription factors after hepatectomy. These include the NF-*κ*B signaling (PHF/NF-*κ*B) and signal transducer and activator of transcription 3 (STAT3) that are responsible for stimulation of the primary growth response or immediate-early genes and are rapidly activated after partial hepatectomy. NF-*κ*B signaling, PHF/NF-*κ*B, is induced within 30 minutes after partial hepatectomy but quickly lost by 1 hour. STAT3 induction is observed within 30 minutes after surgery and peaks between 3 to 5 hours after surgery, extending beyond the immediate-early time period [48]. To determine if the key players in the primary growth response were altered we measured gene expression in livers from sham surgery B6 and 17C-6 mice. The mRNA levels from these livers were quantified for IL-1*β*, interleukin-18 (IL-18), TNF-*α*, NF-Kappa-B Inhibitor-*α* (I*κ*B*α*), IL-6, STAT3, FBJ osteosarcoma oncogene (c-Fos), myelocytomatosis oncogene (c-Myc), C-reactive protein (Crp), and cyclin D1 (Ccnd1) (Figure 7(a)). We found elevated mRNA levels in livers from 17C-6 mice compared to B6 mice for all of the genes measured. To determine if STAT-3 protein levels varied after partial hepatectomy, we measured Stat-3 phosphorylation by Western blot analysis. We found increased Stat-3 phosphorylation at 4 hours after 2/3 partial hepatectomy in 17C-6 mice compared to B6 mice (Figure 7(b)). Thus the livers of 17C-6 mice are primed for primary growth response in liver regeneration.

**Figure 8:**
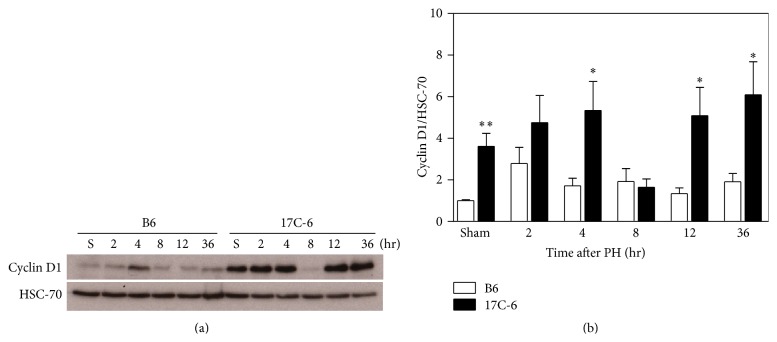
17C-6 mice have increased cyclin D1 protein after 2/3 partial hepatectomy. Two-thirds PH surgeries were performed on 17C-6 congenic and B6 mice. Whole liver protein was isolated and analyzed for expression of cyclin D1 by Western blot. (a) Western blot shown is representative of cyclin D1 after 2/3PH. Western blot was normalized with heat shock cognate-70 (HSC-70) for loading control. (b) Densitometric quantification of Western blots. Values represent the mean ± SEM. ^*∗*^ *P* < 0.05. ^*∗∗*^ *P* < 0.01. Statistics were calculated by two-way ANOVA and Bonferroni correction for multiple testing, *n* = 4–6/group.

**Figure 9:**
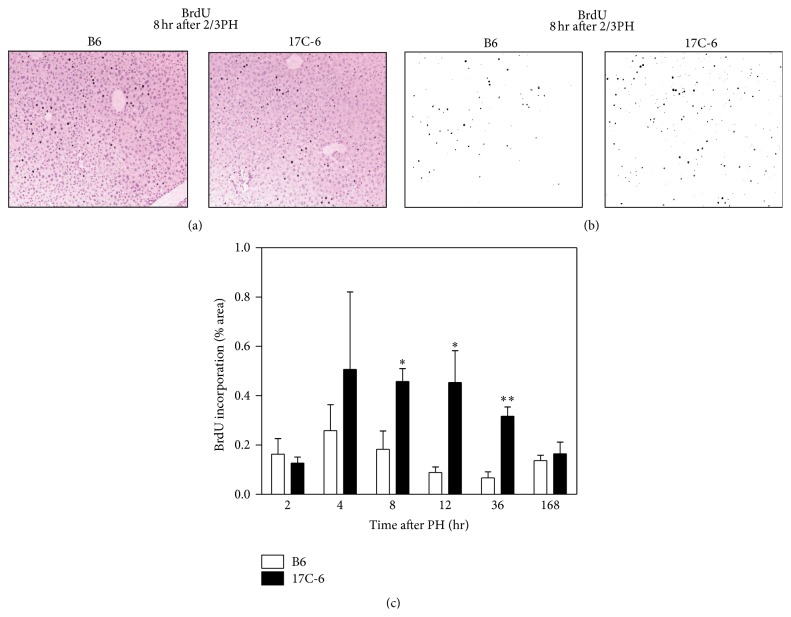
17C-6 mice have increased BrdU incorporation after 2/3 partial hepatectomy. Two-thirds PH surgeries were performed on 17C-6 congenic and B6 mice. BrdU labeling reagent was administered via intraperitoneal injection 2 hours before sacrifice. Liver sections were fixed, mounted, and stained using BrdU detection reagent. (a) Images representative of 8 hours after 2/3PH with positively stained cells counterstained with H&E. (b) Images representative of 8 hours after 2/3PH showing only positively stained cells. (c) The average of four image zones was used to calculate percent (%) area BrdU incorporated per mouse liver. Values represent the mean ± SEM. ^*∗*^ *P* < 0.05. ^*∗∗*^ *P* < 0.01. Statistics were calculated by two-way ANOVA and Bonferroni correction for multiple testing, *n* = 4–6/group.

To test their liver regeneration capacity we measured cyclin D1 protein content in the liver of B6 and 17C-6 mice after 2/3PH. The amount of cyclin D1 is variable over time with a decrease in cyclin D1 at 8 hours, which was shared by both genotypes. However, the 17C-6 mice had significantly greater cyclin D1 levels compared to B6 mice at sham and 4-, 12-, and 36-hour time points (Figure 8). Cyclin D1 is a prominent regulator of the cell cycle at the G1/S phase transition and an indicator of cell mitosis. To quantitatively measure the proliferation of cells within the liver at various time points following 2/3PH, we measured BrdU incorporation into the newly synthesized DNA of replicating cells. The images obtained from microscopy were quantified as percent positive area for BrdU staining using ImageJ Software (Figure 9). This data showed increased positive staining of cells for BrdU for 17C-6 mice over B6 mice beginning at the 8-hour time point and continuing to 12- and 36-hour time points. 17C-6 mice had increased positive staining for BrdU between 4 and 36 hours after surgery. B6 mice showed no increase in positive BrdU staining at any time point measured. This is consistent with data in Figures 6 and 8 as they indicate the majority of liver mass regeneration in the B6 mouse occurs between the 36 hours and 7 days after 2/3PH. The 17C-6 mice have markedly higher hepatic cell proliferation 8 hours after 2/3 partial hepatectomy, lasting at least until 36 hours after surgery when compared to B6 mice. This supports that 17C-6 mice have the capability for an early cell proliferative response during the recovery after 2/3PH (Figure 9).

**Figure 10:**
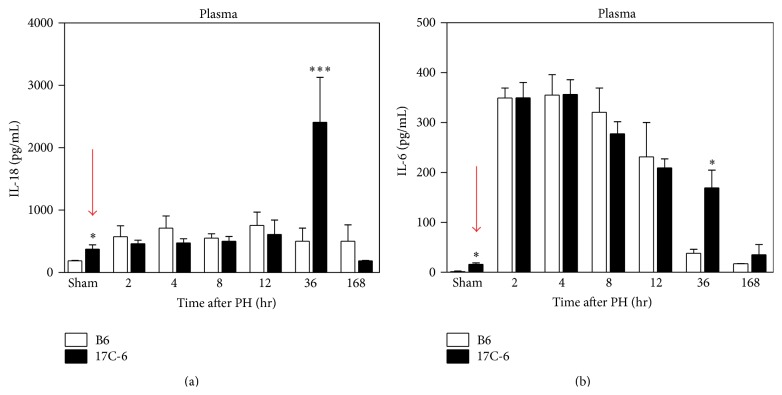
17C-6 mice have increased plasma IL-18 and IL-6 after 2/3 partial hepatectomy. Two-thirds PH surgeries were performed on 17C-6 congenic and B6 mice and their blood plasma was collected. (a) IL-18 and (b) IL-6 were measured by ELISA. Red arrows draw attention to differences at sham time point. Values represent the mean ± SEM. ^*∗*^ *P* < 0.05. ^*∗∗∗*^ *P* < 0.001. Statistics were calculated by two-way ANOVA and Bonferroni correction for multiple testing, *n* = 4–6/group.

To determine if the altered Nlrc4 inflammasome activity impacts circulating inflammatory cytokine levels during liver regeneration, we measured cytokine IL-18 in the plasma. Plasma IL-18 levels were unchanged after 2/3PH in both B6 and 17C-6 except at the sham and 36-hour time points. At sham, 17C-6 mice have a 2-fold increase over B6 mice and at 36 hours 17C-6 have nearly a 5-fold increase over B6 (Figure 10(a)). IL-18 is a potent inducer of IL-6 in murine peritoneal macrophages [49] and the role of IL-6 in liver regeneration is well established [21]. Therefore, we measured plasma IL-6 levels in B6 and 17C-6 mice after 2/3PH. Plasma IL-6 levels were statistically increased in the sham surgery 17C-6 mice compared to B6 mice. IL-6 plasma concentration increased in both B6 and 17C-6 at 2 hours after 2/3PH with no difference between genotypes. The IL-6 levels gradually decline over the course of 12 hours after surgery. However, the 17C-6 mice maintained elevated IL-6 at the 36-hour time point. By 168 hours (7 days) after surgery both genotypes return to baseline (Figure 10(b)).

**Figure 11:**
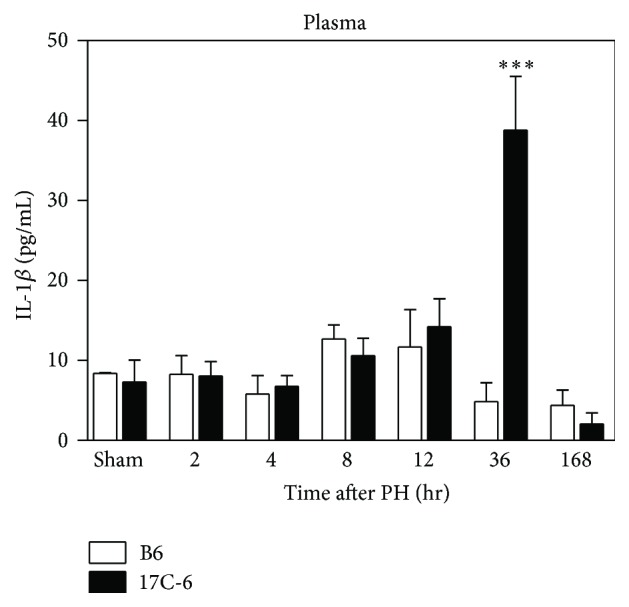
17C-6 mice have increased plasma IL-1*β* after 2/3 partial hepatectomy. Two-thirds PH surgeries were performed on 17C-6 congenic and B6 mice and their blood plasma was collected. IL-1*β* was measured by ELISA. Values represent the mean ± SEM. ^*∗∗∗*^ *P* < 0.001. Statistics were calculated by two-way ANOVA and Bonferroni correction for multiple testing, *n* = 4–6/group.

A functional product of Nlrc4 inflammasome activation is production and secretion of both proinflammatory cytokines IL-18 and IL-1*β*; therefore IL-1*β* was also measured in the plasma. We found no differences in both B6 and 17C-6 treated mice except at the 36-hour time point after surgery. At 36 hours 17C-6 has nearly an 8-fold increase over B6. By 168 hours (7 days) however, both animal groups have returned to basal plasma IL-1*β* levels (Figure 11).

### 3.6. Acute CCl_4_ Treatment: 17C-6 Mice Exhibit Increased Mitosis after Acute Injury

**Figure 12:**
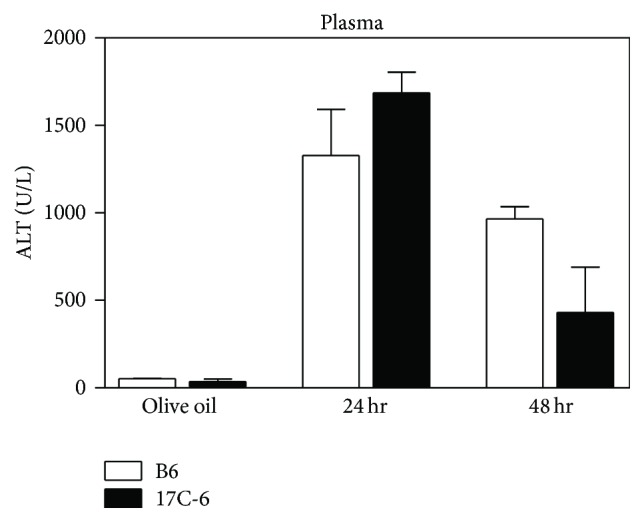
17C-6 congenic mice have similar liver damage to B6 after a single dose of CCl_4_. B6 and 17C-6 mice were given a single intraperitoneal injection of hepatotoxin CCl_4_ or olive oil vehicle. CCl_4_ mice were sacrificed 24 or 48 hours after injection and their blood plasma was collected. Olive oil controls were sacrificed 48 hours after injection. Plasma ALT levels were measured by enzymatic assay. Values represent the mean ± SEM. Statistics were calculated by two-way ANOVA and Bonferroni correction for multiple testing, *n* = 4–6/group.

The ability of 17C-6 mice to resist CCl_4_-induced liver fibrosis could be a result of either decreased hepatic injury or increased capacity to restore hepatic function after injury. To further test restoration of hepatic induced injury by CCl_4_, B6 and 17C-6 mice were administered a single dose of CCl_4_ and allowed to recover for 24 and 48 hours. Plasma alanine aminotransferase (ALT) levels were measured as an indicator of hepatocyte injury. We found that plasma ALT levels peaked at 24 hours (1500 U/L) for both groups with no statistical difference. ALT levels began to recover at 48 hours for both groups, with B6 averaging 965 U/L and 17C-6 averaging 431 U/L, respectively. There were no statistical differences between B6 and 17C-6 at 48 hours (Figure 12). This data indicates that the degree of injury in the B6 and 17C-6 livers after acute CCl_4_ is similar.

**Figure 13:**
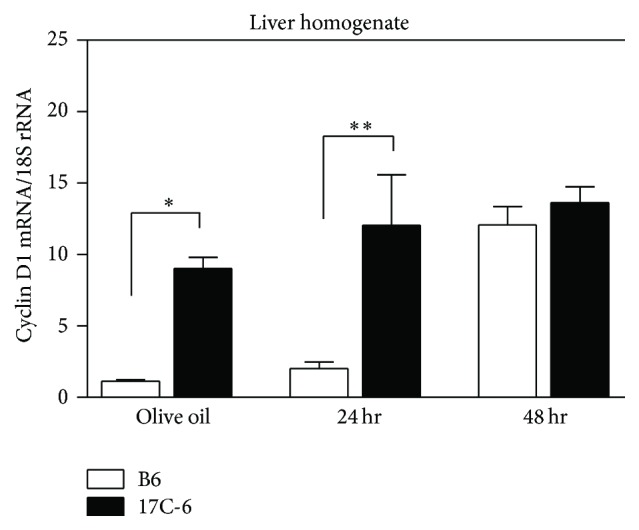
17C-6 congenic mice have increased levels of cyclin D1 after a single dose of CCl_4_. B6 and 17C-6 mice were given a one-time intraperitoneal injection of hepatotoxin CCl_4_ or olive oil vehicle. CCl_4_ mice were sacrificed 24 or 48 hours after injection and their liver tissue was collected. Olive oil controls were sacrificed 48 hours after injection. Total RNA was isolated from liver tissue. Cyclin D1 mRNA was quantified by qPCR analysis and normalized with 18S rRNA. Values represent the mean ± SEM. ^*∗*^ *P* < 0.05. ^*∗∗*^ *P* < 0.01. Statistics were calculated by two-way ANOVA and Bonferroni correction for multiple testing, *n* = 3/group. No statistical differences were found.

Congenic mouse 17C-6 has shown remarkable resistance to hepatotoxin CCl_4_-induced fibrosis but has similar hepatocyte damage after acute exposure to this toxin. In order to replace the hepatic cells lost to this damage, existing cells must proliferate to restore hepatic function. To test cellular proliferation we measured the content of cyclin D1 mRNA, a well-established marker of cellular mitosis, in B6 and 17C-6 mice given a single injection of CCl_4_ (Figure 13). In olive oil controls we found a 9-fold increase of hepatic cyclin D1 mRNA. By 24 hours the 17C-6 mice showed a 10-fold increase in hepatic cyclin D1 mRNA compared to B6 mice. By 48 hours the level of cyclin D1 mRNA is similar in both B6 and 17C-6 mice. Therefore, the hepatic cells in 17C-6 mice are continually in a proliferative phase, while the B6 mice match the proliferative capacity of 17C-6 at 48 hours after hepatotoxin CCl_4_ injection.

## 4. Discussion

As NAFLD progresses to the stage of fibrosis, the excessive deposition of extracellular matrix proteins modifies the hepatic architecture. This modification inhibits normal portal blood flow and limits liver function [50]. Fibrosis is the consequence of unrelenting wound-healing response after repeated injury to the liver. We previously have shown that 17C-6 animals were resistant to CCl_4_-induced fibrosis [33]. Here we have established that 17C-6 mice have an increased regenerative liver capacity after 2/3PH (Figures 6–11). By stimulating hepatocytes to promote cell survival and proliferation (through Nlrc4 and inflammatory cytokines), the liver is capable of withstanding repeated trauma with a superior wound-healing response. This may limit the fibrotic response typical of repeated liver trauma that leads to cirrhosis and ultimately liver failure.

When genetic polymorphisms are present within the coding or regulatory regions of a gene there is a potential for dysregulation of gene function leading to disease. We have identified two polymorphisms affecting regulation of the Nlrc4 gene. The first was an unidentified SNP (rs74459439-T) situated 331 bases upstream of the Nlrc4 transcriptional start site. Using transcription factor binding identification software we were able to identify this variable region of the promoter as a potential binding site for transcription factor Cdx-1. ChIP analysis showed no interaction between Cdx-1 and the B6 sequence at the SNP location (Figure 2). However, Cdx-1 did bind to other Cdx-1 binding sites within the Nlrc4 promoter of the B6 mouse (Figure 2, Supplemental Figure 1). Gene expression analysis also showed increased Nlrc4 expression when a promoter contained the SNP from the A/J allele for Nlrc4 (17C-6 mice) (Figure 3). This indicates that the single base pair deletion in the A/J allele for Nlrc4 promoter resulted in increased gene expression in RAW 264.7 cells. Overexpression of Nlrc4 in human cells lines has shown increased inflammatory response to* Salmonella* infection. Also, Nlrc4 overexpression leads to homooligomerization which results in mild caspase-1 activation independent of bacterial flagellin stimuli [29, 51].

A second SNP was located within the coding region of Nlrc4, in exon 5 [52]. The A/J allele contains the SNP which resulted in a nonsynonymous mutation leading to an amino acid substitution (I756V), isoleucine (B6) substituted for valine (17C-6). This mutation occurs within the leucine rich repeat domain in Nlrc4, a domain suggested to sequester Nlrc4 in a monomeric inactivated state. Others have shown that a deletion in this region of the protein results in a constitutively active Nlrc4 and increased processing of IL-1*β* [53, 54]. Our data shows these SNPs display increased processing of IL-1*β* (Figure 5(a)). Together, these two polymorphisms yield a mildly constitutively active Nlrc4 inflammasome in macrophages of the 17C-6 congenic mouse.

Non-caspase-1 processing of IL-1*β* has been documented. Sterile inflammation attributable to IL-1*β* originating from neutrophils causes an increase in IL-6 secretion and acute phase proteins [55]. This response may be blunted using inhibitors of IL-1*β* but not caspase-1, indicating a non-caspase-1 mediated mechanism. In this case neutrophil processing of IL-1*β* is attributed to extracellular processing via proteinase-3 [56]. This proteinase has also been shown to participate in processing of IL-18 [57].

In 2014, two independent groups of investigators published the discovery of different gain-of-function Nlrc4 mutations [58, 59]. The mutations occur in the nucleotide binding pocket of Nlrc4 resulting in constitutive activation. Both mutations produce increased IL-1*β* and IL-18 in serum. The patients have fever, gastrointestinal distress, and splenomegaly in the absence of any detectable infection. The disease was termed Nlrc4-macrophage activation syndrome (Nlrc4-MAS) and shares similarity to mutations in the NLRP3 gene that result in the autoinflammatory disease, neonatal onset multisystem inflammatory disease (NOMID) [60]. Monocytes derived from patients with Nlrc4-MAS show increased IL-1*β* secretion upon LPS stimulation compared to NOMID cells. Nlrc4-MAS monocytes and macrophages show constitutive secretion of IL-18 in absence of stimuli while NOMID cells show no such secretion [58]. Clinicians are able to successfully treat Nlrc4-MAS with IL-1 receptor antagonist (anakinra) [58].

The gold standard to study liver regeneration is 2/3PH. In order for the liver to successfully restore the tissue lost to surgery it must orchestrate a complex endocrine signaling response from multiple cell types, initiating cellular survival and proliferation until original liver mass is restored. When challenged with 2/3PH, 17C-6 mice displayed a remarked increase in regenerative capacity (Figure 6). Others have shown that B6 mice restore the original liver mass within 7–14 days after 2/3PH [61–63], with hepatocyte regeneration reaching a peak between 30 and 60 hours after 2/3PH [64]. Our B6 mice liver regenerative rate is in agreement with previous investigators while 17C-6 mice initiate restoration much sooner. We chose to focus on early time points of hepatic regeneration as we hypothesized that an improved ability to regenerate dead and dying hepatocytes following liver injury may be the mechanism by which the 17C-6 mice are protected from CCl_4_-induced fibrosis.

The acute phase response is a coordinated early defense reaction in the liver responsible for protection from pathogenic infection, repair of damaged tissue, and the restoration of the proinflammatory state in response to infection and/or trauma [65]. The acute phase response is mediated by IL-1*β*, TNF-*α*, and IL-6 cytokines and the production of acute phase proteins [66]. IL-6 deficient mice also display an impaired ability to regenerate their liver after 2/3PH. This impaired regeneration can be rescued with a preoperative exogenous IL-6 treatment [21]. Peters et al. designed an IL-6/sIL-6R fusion protein (Hyper-IL-6) that mimics IL-6 transsignaling by directly stimulating gp130 in the absence of IL-6R. After partial hepatectomy, they demonstrated that Hyper-IL-6, but not IL-6 alone, led to early induction of hepatocyte proliferation implicating IL-6 transsignaling in the regulation of liver regeneration [67]. We hypothesize that 17C-6 mice have increased liver regenerative capacity due to elevated levels of these cytokines, leading to increased liver tissue remodeling and repair.

IL-18 is synthesized as an inactive precursor and must be activated through proteolytic cleavage by caspase-1. Caspase-1 itself must be cleaved by the inflammasome complex to be activated. The IL-18 precursor is constitutively expressed in many cell types including the resident macrophages of the liver, the Kupffer cells [68]. Should Nlrc4 have mild continual activation, it would repeatedly generate an active caspase-1, independent of a stimulatory trigger for the Nlrc4 inflammasome activation [53]. This active caspase-1 would in turn activate the pool of pro-IL-18, thus generating proinflammatory molecules that may amplify into a full inflammatory response mediated by the generation of more pro-IL-18, as well as pro-IL-1*β* and TNF-*α*. We have shown an increase in gene expression of all three of these cytokines after sham 2/3PH (Figure 7(a)), as well as increased plasma concentrations of IL-18 after sham 2/3PH (Figure 10(a)). For IL-18 to amplify the production of inflammatory cytokines it must bind to its membrane bound receptor and signal through the transcription factor complex NF-*κ*B. NF-*κ*B activity may be measured transcriptionally as it promotes the transcription of its inhibitor I*κ*B*α* to serve as negative feedback [69]. Within 17C-6 mice we found a statistical increase in mRNA levels of IL-1*β*, IL-18, TNF-*α*, and I*κ*B*α* relative to B6 mice (Figure 7(a)). Interestingly, IL-1*β* protein levels in plasma do not increase until hour 36 after surgery in 17C-6 mice. We postulate that this 36-hour lag period may be attributed to the time needed to amplify the localized inflammatory response and secrete enough IL-1*β* that is detectable in the plasma.

The replacement of hepatic cells lost to infection, trauma, and inflammation is primarily mediated through IL-1 family cytokines, TNF-*α*, and IL-6 (all transcriptional targets of NF-*κ*B [70–73]) through their induction of the inflammatory acute phase response. A key regulator of hepatocyte regeneration, IL-6, is produced as a consequence of the inflammatory signaling cascade initiated by the Nlrc4 inflammasome. Downstream targets of IL-6 signaling (c-Fos, c-Myc, Crp, and cyclin D1) participate in hepatocyte survival and proliferation. We demonstrated that 17C-6 mice have increased IL-6 transcription levels in the sham surgery liver as well as systemically increased plasma IL-6. Therefore IL-6 carries out signaling from the macrophage to the hepatocyte by interaction with the membrane receptor complex IL-6r/Gp130 in the hepatocyte. Subsequent signaling through STAT3 results in transcriptional activation of acute phase response proteins such as Crp, generation of cell-cycle regulators including cyclin D1, as well as transcription factors in control of cellular differentiation and cycle progression, c-Myc and c-Fos [74–76]. 17C-6 mice have increased mRNA concentrations for STAT3, cyclin D1, c-Fos, and c-Myc in liver tissue obtained in the sham surgery and increased phosphorylation of STAT3 after PH (Figure 7(b)). This indicates a strong proliferative signal within the hepatocytes, even before the liver experiences trauma. The cells are primed for a quick reaction to liver damage with an increased ability to resolve this damage, diminishing the overall effects of hepatic trauma. This potentially contributes to the decreased susceptibility to fibrosis development seen in congenic mouse 17C-6.

Previously we have published that 17C-6 mice were resistant to CCl_4_-induced liver fibrosis [77]. For 17C-6 to have a marked decrease in accumulation of extracellular matrix proteins (fibrosis) the mice must have either reduced hepatotoxin-induced injury or an amplified ability to regenerate or increased ECM turnover as well as other possibilities. To fully understand this decrease in fibrosis susceptibility, B6 and 17C-6 mice were acutely exposed to CCl_4_. 17C-6 mice given a single dose model of carbon tetrachloride displayed hepatic injury comparable to B6 mice. However we found increased hepatic mitosis in 17C-6 mice (Figure 13).

**Figure 14:**
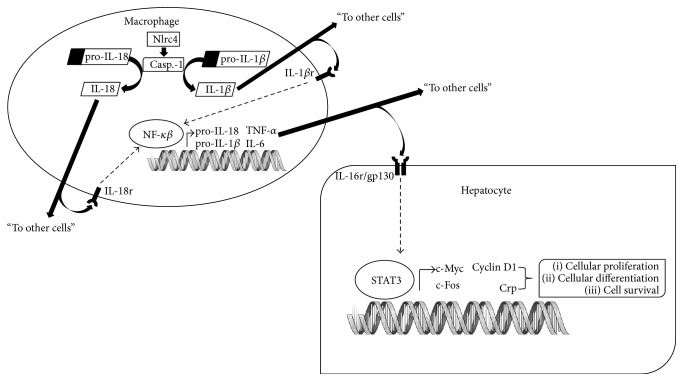
Proposed model of Nlrc4 induced liver regeneration. In the basal state the 17C-6 mice have increased plasma IL-18 due to the chronic activation of Nlrc4. IL-18 binds to its receptor in the KC to activate NF-*κ*B signaling which in turn increases expression of IL-6, TNF-*α*, IL-18, and IL-1*β*. The elevated IL-6 binds to IL-6 receptor/gp130 in the liver and activates JAK/STAT signaling. The activated STAT3 initiates increased cyclin D1, Crp, c-Myc, and c-Fos which in turn increases the rate of hepatic cell proliferation, differentiation, and survival.

In Figure 14 we propose a model for the increased regenerative capacity in liver from 17C-6 mice. The A/J allele for Nlrc4 has two SNPs that result in chronic low level activation of Nlrc4 in the Kupffer cells. In the basal state the 17C-6 mice secrete increased IL-18 due to the chronic activation of Nlrc4 (Figure 10(a)). IL-18 binds to its receptor in the Kupffer cell and initiates signaling to activate NF-*κ*B signaling which in turn increases expression of IL-6, TNF-*α*, IL-18, and IL-1*β*. The elevated IL-6 binds to IL-6 receptor/gp130 in the liver and activates JAK/STAT signaling. The activated STAT3 induces cyclin D1, Crp, c-Myc, and c-Fos transcription, which together, increase the rate of hepatic cell proliferation, differentiation, and survival.

By characterizing the Nlrc4 inflammasome and its association to liver regeneration we have potentially provided new insights for treatment of liver disease. Here we have identified that an Nlrc4 inflammasome-driven production of inflammatory cytokine signaling leads to a coordinated hepatoprotective response to CCl_4_-induced and hepatectomy-induced liver damage. The ability of inflammatory cytokines TNF-*α*, IL-1*β*, and IL-18 to stimulate hepatocyte proliferation, mediated through IL-6, permits for a flexible restoration network to repair liver tissue after trauma. Further investigation is necessary to research the signaling pathways responsible for hepatic regeneration, specifically their intercellular signaling cascades and subsequent cellular responses.

## 5. Conclusions

Taken together, these data demonstrate that the Nlrc4 inflammasome regulates liver regeneration. This study validates that constitutive activation of the inflammasome to produce mature interleukins IL-18 and IL-1*β* within the liver leads to the increased production and secretion of IL-6, a key regulator of liver regeneration. This mild, continuous inflammatory response was shown to diminish the development of hepatotoxin-induced fibrosis as well as facilitate the enhanced regeneration of liver mass after hepatectomy. The ability to promote healing within a damaged liver has direct clinical implications for the prevention and/or treatment of diseases of the liver.

## Supplementary Material

The supplementary data contains ChIP analysis of Cdx-1 binding sites to the mouse Nlrc4 promoter, the primer sequence used for the ChIP analysis, and the primer sequences for real-time quantitative PCR (qPCR).
