# Supplementary material for: Constitutive Activation of the Nlrc4 Inflammasome Prevents Hepatic Fibrosis and Promotes Hepatic Regeneration after Partial Hepatectomy
Source: Mediators Inflamm. 2015 Nov 9;2015:909827. doi: 10.1155/2015/909827 (PMC4655266; doi:10.1155/2015/909827)
Supplement: Supplementary file 1 — The supplementary data contains ChIP analysis of Cdx-1 binding sites to the mouse Nlrc4 promoter, the primer sequence used for the ChIP analysis, and the primer sequences for real-time quantitative PCR (qPCR). [file 909827.f1.pdf]

Supplemental Files:

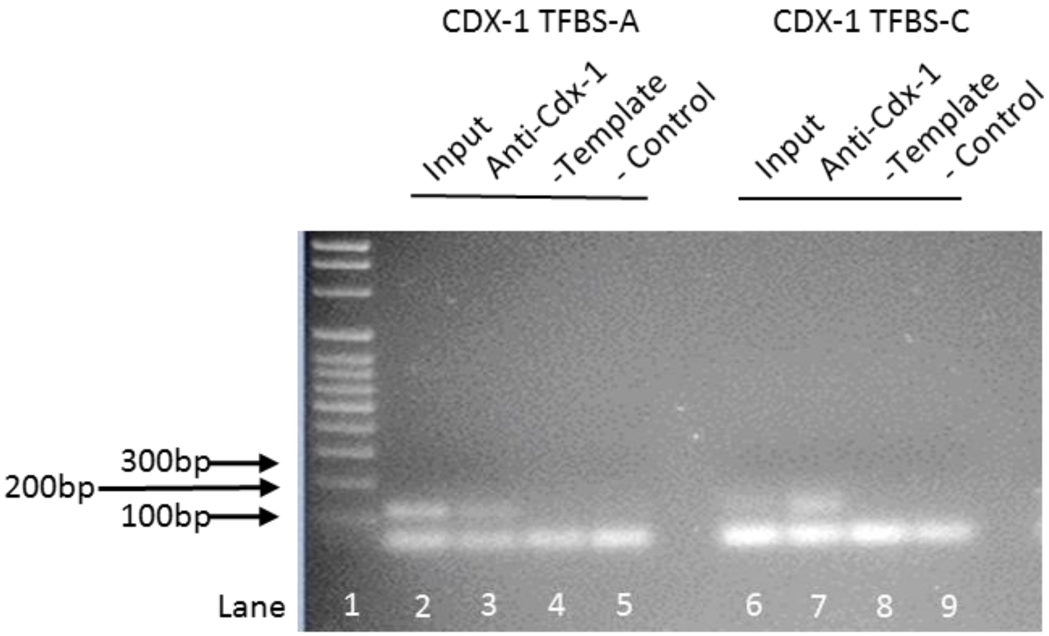

Supplemental Figure 1. Cdx-1 binds to the mouse Nlrc4 promoter. ChIP analysis of DNA in cross-linked chromatin from RAW264.7 cells. DNA fragments were precipitated using an anti-Cdx-1 antibody. Lanes 2-5: Primer sequence targeting TFBS-A using RT-PCR. Lanes 6-9: Primer sequence targeting TFBS-C using RT-PCR. Lane 1: DNA Ladder. Lane 2: 1% starting chromatin input. Lane 3: DNA precipitated with anti-Cdx-1 antibody Lane 4: Non-template control. Lane 5: negative control using anti-GFP antibody. Lane 6: 1% starting chromatin input. Lane 7: DNA precipitated with anti-Cdx-1 antibody Lane 8: Non-template control. Lane 9: negative control using anti-GFP antibody. RT-PCR products were visualized using a 2% agarose gel.

Primer Label	Forward Sequence (5'-3')	Reverse Sequence (5'-3')	Amplicon size
CDX-1 TFBS-A	ctctccagcccctttgaact	atcttgagccatctgggttg	121
CDX-1 TFBS-B	cccaatgtttccaccaatgt	ccacagagacccacctgact	150
CDX-1 TFBS-C	ctcaggaggcagagtcaggt	tgttttgttttgttttgggaca	117
CDX-1 TFBS-D	cattccctttgcttggagaa	gatgccctcttctggtgtgt	150

Supplemental Table 1. Primer sequences for ChIP. ChIP primer sets were custom designed using Primer3Plus PCR primer design tool (http://sourceforge.net/projects/primer3/).

Primer Label	Official Symbol	Forward Sequence (5'-3')	Reverse Sequence (5'-3')	Amplicon size
18S	Rn18s	agaaacggctaccacatccaa	gactcattccaattacagggc	123
Cyclin D1	Ccnd1	cgtggcctctaagatgaagga	cgggccggatagagttgt	80
IL-1β	Il1b	caggcaggcagtatcactca	tgtcctcatcctggaaggtc	86
IL-18	Il18	tccatgctttctggactcct	ggctttctttgtcctgatgc	89
IL-6	Il6	gttctctgggaaatcgtgga	cagaattgccattgcacaac	52
TNF-α	Tnf	agccccccagtctgtatcctt	gagttggaccctgagccata	58
IκBα	Nfkbia	ctccagatgctacccgagag	tagggcagctcatcctctgt	77
Stat3	Stat3	cagggtgtcagatcacatgg	tagccagacccagaaggaga	77
c-Fos	Fos	cgggtttcaacgccgacta	ttggcactagagacggacaga	166
c-Myc	Myc	gagtgcattgacccctcagt	gaatcggacgaggtacagga	80
Crp	Crp	tctgcacaagggctacactg	aagtcaccgccatacgagtc	78

Supplemental Table 2. Primer sequences for qPCR. qPCR primer sets were custom designed using Primer3Plus PCR primer design tool (http://sourceforge.net/projects/primer3/) with design optimization for qPCR application.
